# Observations of Mammalian Predation on Pacific Sleeper Sharks in Southeast Alaska

**DOI:** 10.1002/ece3.73854

**Published:** 2026-11-05

**Authors:** Cindy A. Tribuzio, John Moran

**Affiliations:** ^1^ Auke Bay Laboratories Alaska Fisheries Science Center, National Marine Fisheries Service, NOAA Juneau Alaska USA

**Keywords:** natural mortality, offshore killer whales, Steller sea lions

## Abstract

The predator–prey relationship between marine mammals and sharks may not be as well defined as originally believed. Both groups can be either predator or prey. In the case of the Pacific sleeper shark, previous studies have documented the sharks predating upon juvenile Steller sea lions and scavenging whale falls, while other studies have reported offshore killer whales predating upon Pacific sleeper shark. Here, we report two observations of likely marine mammal predation upon Pacific sleeper sharks in Southeast Alaska; one of Steller sea lions actively predating upon an approximately 2 m shark; and the other a stranded carcass of a 3 m shark likely predated by killer whales. The observations reported herein are unique in that Steller sea lions have not been documented to predate upon Pacific sleeper sharks, and previous reports of killer whale predation upon Pacific sleeper sharks have been near open ocean passages.

## Graphical abstract

The predator–prey relationship between marine mammals and large sharks is complicated. We report on observations of likely predation by Stellar sea lions and killer whales on Pacific sleeper sharks in Southeast Alaska.

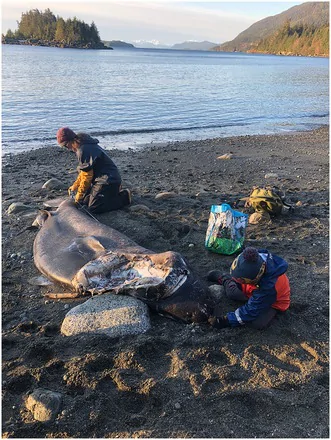

The Pacific sleeper shark ( *Somniosus pacificus* , Figure 1) is a large bodied shark species that is relatively common in nearshore, high latitude waters. The species is widely distributed across the North Pacific Ocean with reported occurrences extending into the South Pacific and Southern Oceans (Claassens et al. 2023, Matta et al. 2024, Rigby et al. 2021, Tian et al. 2024). Pacific sleeper sharks also occupy a wide depth range, from depths of at least 2000 m to surface waters at high latitudes (Hulbert et al. 2006, Wang et al. 2024). Within Alaska, fishery‐dependent (i.e., catch records) and ‐independent (e.g., surveys and research) data show that Pacific sleeper sharks can be found in waters very close to shore and throughout the water column, specifically in the fjords of Prince William Sound and Southeast Alaska (Bishop et al. 2026, Courtney and Hulbert 2007, Hulbert et al. 2006, Matta et al. 2024, Tribuzio et al. 2022, Tribuzio unpublished data and anecdotal reports). The species is considered one of the most vulnerable fish stocks in Alaska waters due to its slow growth and low productivity and was recently classified as “Near Threatened” globally by the IUCN (Ormseth and Spencer 2011; Rigby et al. 2021; Tribuzio et al. 2022). Pacific sleeper sharks are not targeted in Alaska, but incidental catch in target fisheries does occur. The catch limits for fisheries are set based on historical catch, as there is not enough information available to evaluate the status of the stock or population trends (Tribuzio et al. 2022). Natural mortality, a common proxy for inferring the productivity of a stock, is the population's rate of death from all non‐fishing causes, including predation, and is useful for guiding harvest advice outside of the stock assessment.

**FIGURE 1:**
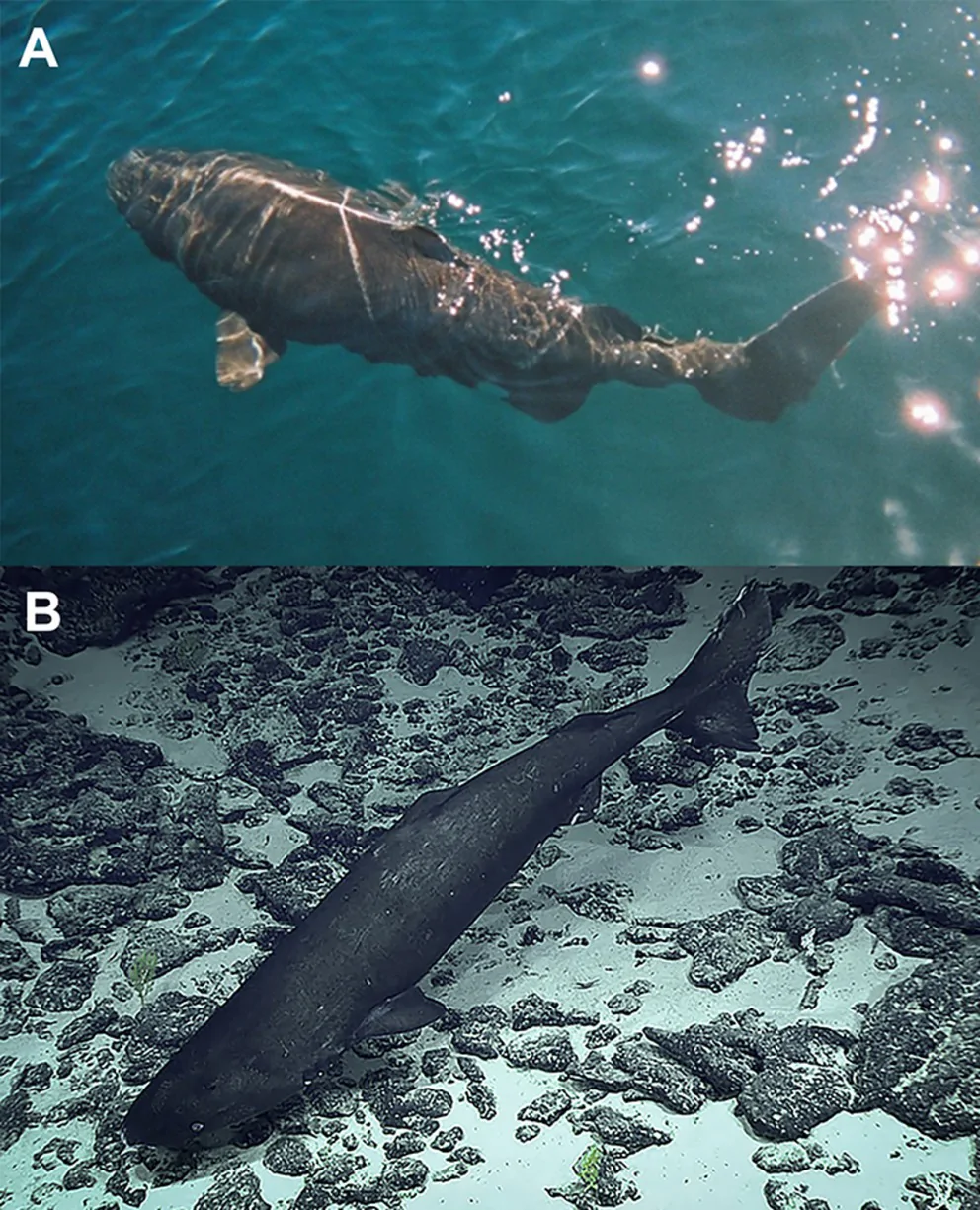
(A) Pacific sleeper shark ( *Somniosus pacificus* ) swimming at the surface after release from survey gear. Credit: International Pacific Halibut Commission. (B) Pacific sleeper shark photographed at 3125 ft depth by the remotely operated vehicle, Deep Discoverer. Credit: NOAA Office of Ocean Exploration and Research.

To inform uncertainty in natural mortality parameter assumptions, we describe two predation events observed in Southeast Alaska that highlight trophic interactions between sharks and marine mammals. First, we report on Steller sea lions ( *Eumetopias jubatus* ) preying on a Pacific sleeper shark. Recent studies have investigated predation on Steller sea lions by Pacific sleeper sharks (Bishop et al. 2019; Frid et al. 2009; Horning and Mellish 2014); however, we found evidence of the reverse scenario. Second, we describe an incident that was likely an offshore killer whale ( *Orcinus orca* ) predation event. Offshore killer whales are an ecotype that inhabit the northeastern Pacific Ocean (Dalheim et al. 2008), specializing in feeding on elasmobranchs with Pacific sleeper sharks comprising as much as 45% of their diet (Ford et al. 2011, Wright et al. 2025). While all three of these species overlap in Alaska, the extent of the predation impact on the mortality rates and behavior of Pacific sleeper sharks is unknown.

On June 4, 2010, during a routine humpback whale survey (NMFS Permit No. 24378), shortly after noon in North Pass (58.477 N, −134.932 W, Figure 2), a narrow body of water between Lincoln and Shelter Islands about 36 km northeast of Juneau, Alaska, we observed a group of four Steller sea lions (observed under NMFS Permit No. 18537) harassing an approximately 2 m long Pacific sleeper shark. Adult male Steller sea lions can reach 1120 kg and a length of 3.25 m (Loughlin 1998), are highly maneuverable, armed with large canine teeth, and can subdue prey as large as harbor seals (Mathews and Adkison 2010). Steller sea lions are common in Southeast Alaska. The population was increasing at 4.25% from 1990 to 2017 but has appeared to level out since 2017, with the Lynn Canal sub‐population estimated to be 570 non‐pups (Young et al. 2024). In this observation, the group of Steller sea lions consisted of at least one adult male and three other large individuals. Upon the initial sighting, the shark appeared to be alive but severely injured. The group of Steller sea lions appeared to be keeping the shark at the surface, dragging it by the caudal fin. Within 20 min, the shark's liver was removed and partially consumed (Figure 3). The remainder of the carcass was not seen again. The water depth averaged 135 m during the encounter, well within the maximum observed dive depths for adult Steller sea lions in southeastern Alaska (Rehberg et al. 2009), suggesting that the shark could have been captured anywhere in the water column.

**FIGURE 2:**
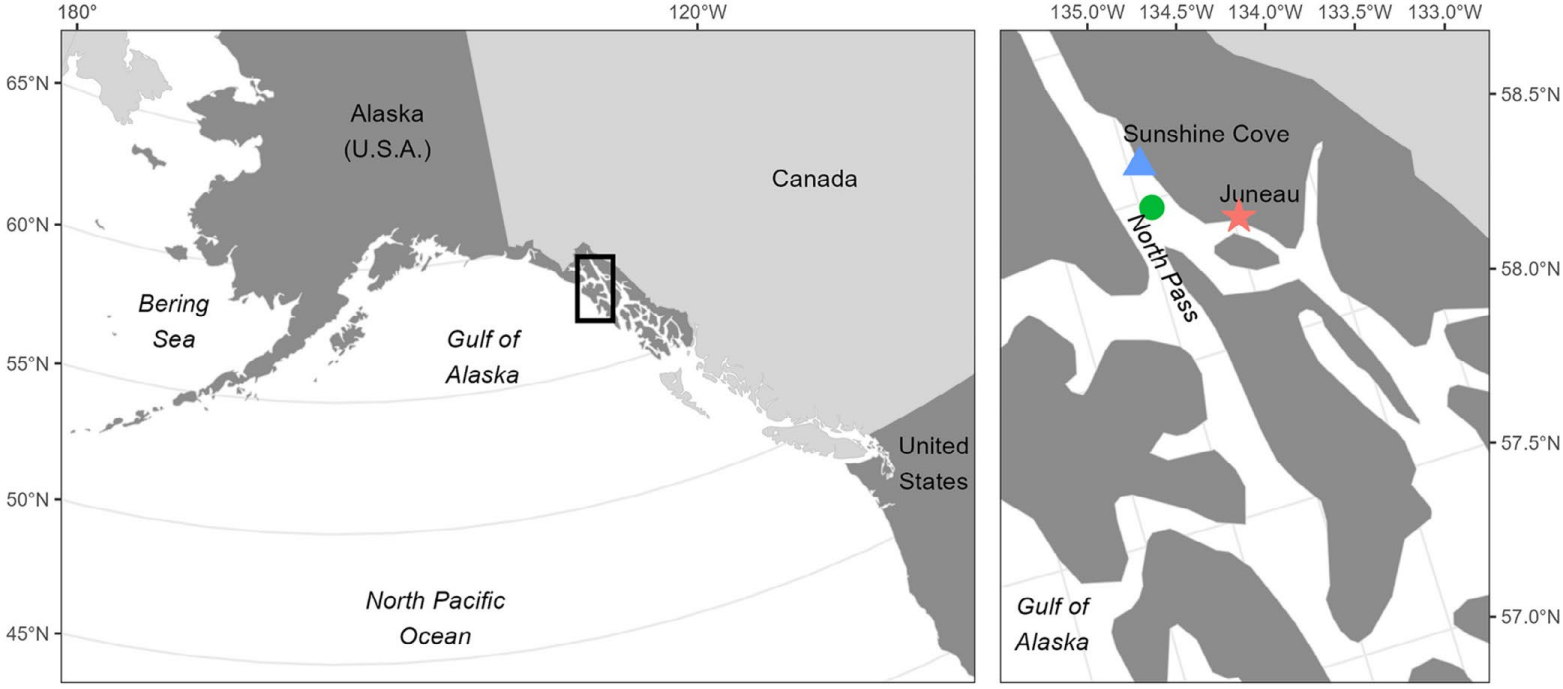
Left panel shows map of the North Pacific Ocean with a black box around the study area. Right panel shows the locations of the predation events on Pacific sleeper sharks ( *Somniosus pacificus* ) observed in North Pass (green circle) and stranded in Sunshine Cove (blue triangle).

**FIGURE 3:**
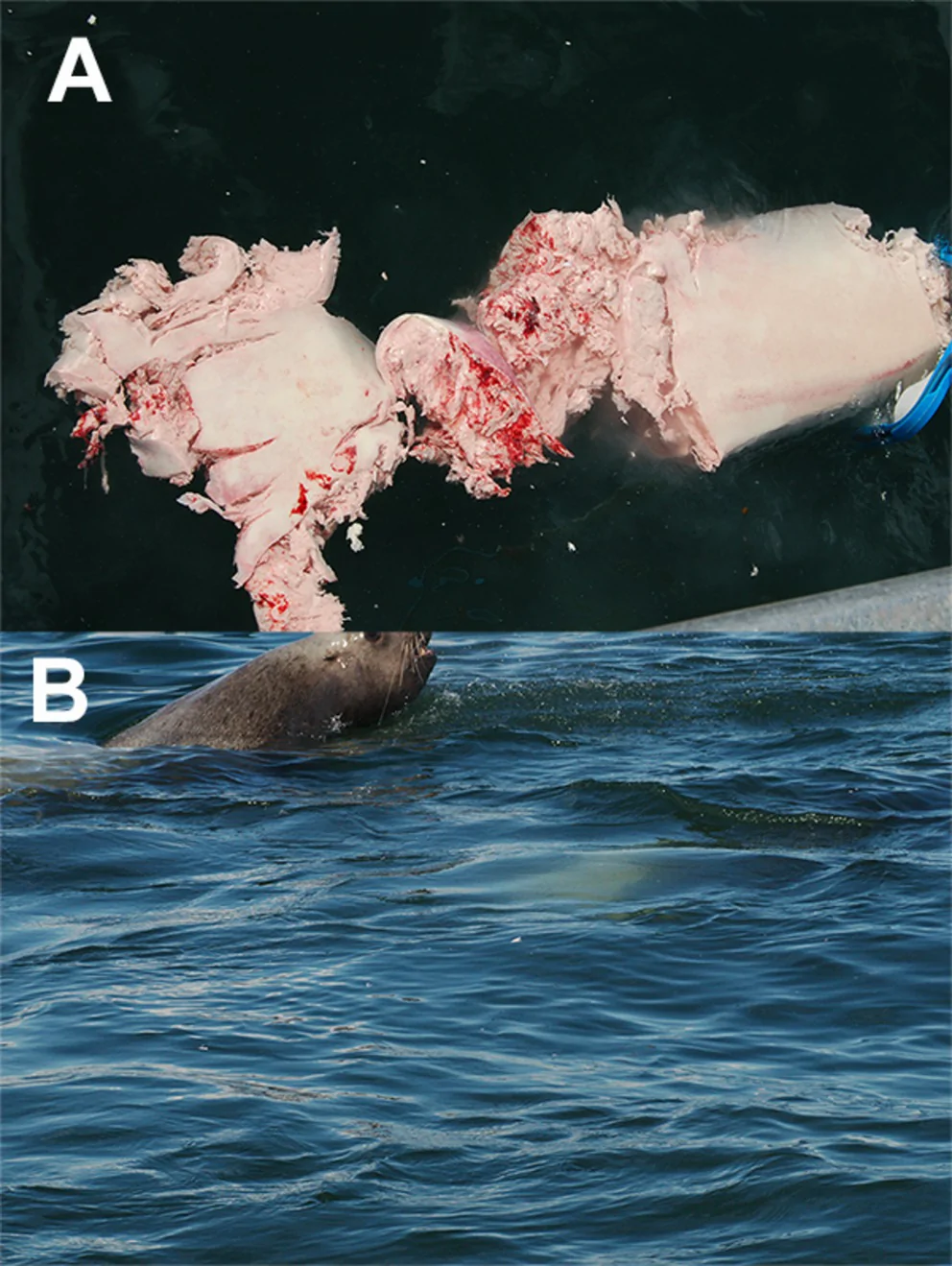
(A) Large fragment of Pacific sleeper shark ( *Somniosus pacificus* ) liver found floating after an observed predation event on June 4, 2010. (B) Steller sea lion ( *Eumetopias jubatus* ) swimming in the vicinity of a fragment of the shark liver (bottom). These observations occurred under NMFS Permit No. 18537.

The second encounter was the carcass of a Pacific sleeper shark found washed ashore in Sunshine Cove (58.609 N, −134.931 W), a small embayment about 56 km north of Juneau, Alaska on the morning of March 7, 2022 (Figure 2). A member of the public observed the shark and notified National Marine Fisheries Service staff. A necropsy team was able to access the carcass shortly thereafter. There was no evidence of significant bird predation (e.g., undamaged eyes). The presence of blood in and around the body and a lack of decomposition suggested that it was a recent stranding. The specimen was a 2.6 m, pre‐caudal length (measured in a straight line from the tip of the snout to the dorsal pre‐caudal notch) female Pacific sleeper shark. The pre‐caudal length results in an estimated natural total length of 3 m (with the caudal fin in the natural position). Total length was not measured in the field, and the estimated total length is based on length transition equations from Matta et al. (2024). The female was in the early stages of maturity, where the ovaries contained identifiable small granular ova, but the oviducts and uteri were not easily differentiated (Figure 4). The shark was lying on its dorsal right side with severe trauma to the pharynx region and a deflated appearance (Figure 5). The lower jaw was intact, but the basihyal (tongue‐like structure), skin, and portions of the gills had been removed between the lower jaw and the third gill slit. The edges of the injury were ragged, consistent with being torn (Figure 6A). There were also external puncture wounds observed on the left ventral portion of the body, just anterior to the pelvic fins (Figure 6B). The internal cavity had evidence of several injuries. The liver, heart, and much of the digestive tract had been removed, and the interior wall of the gut was bruised (Figure 7A). There were internal puncture wounds, which matched the observed exterior punctures, as well as internal punctures on the right ventral side (Figure 7B). The spacing of these parallel injuries was 4.5–5.0 cm, consistent with killer whale tooth marks (George et al. 1994). It was not possible to roll the shark over to look for corresponding external punctures on the right side.

**FIGURE 4:**
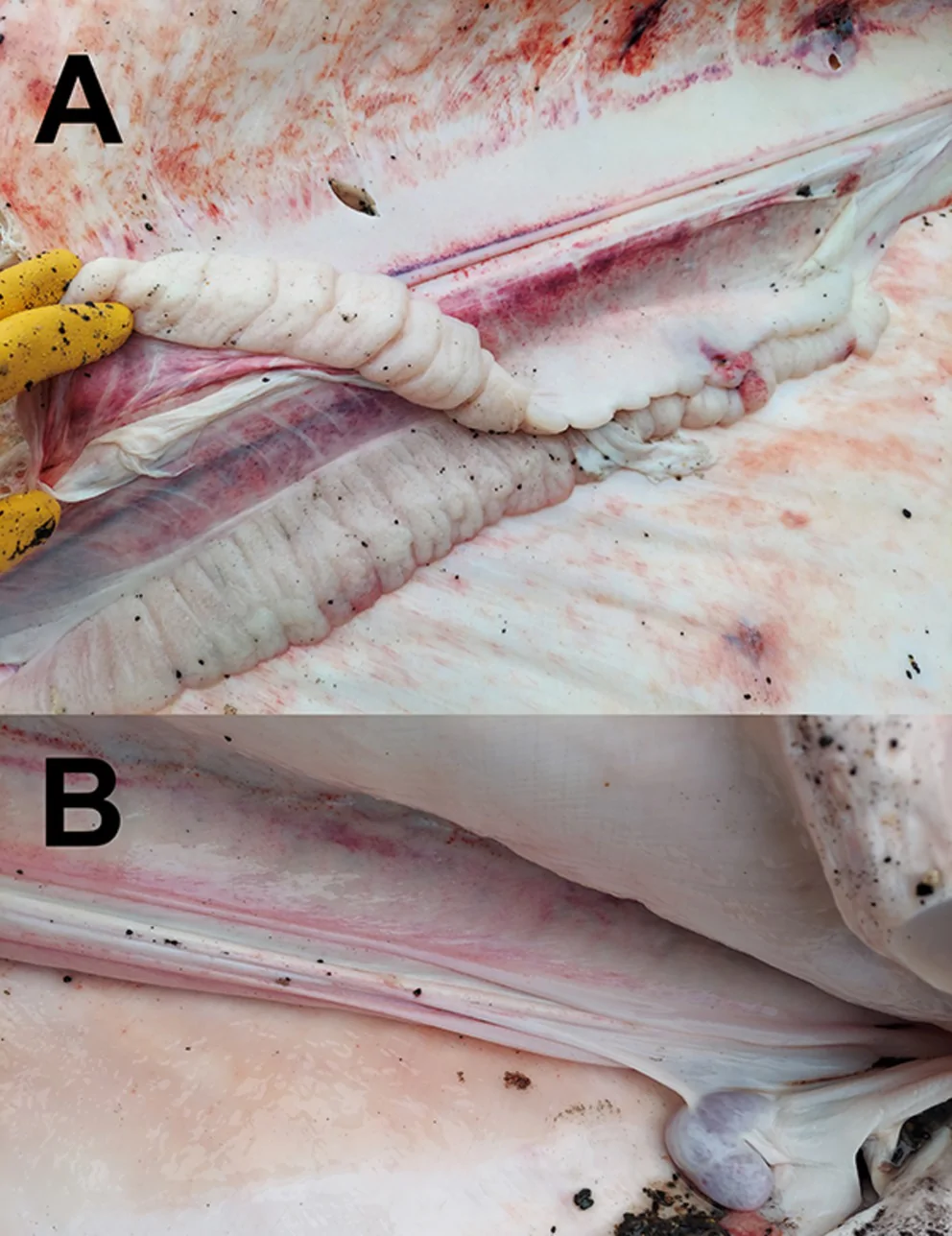
Reproductive tract of the female Pacific sleeper shark ( *Somniosus pacificus* ) found deceased in Sunshine Cove. (A) Ovaries with granular ova, suggesting early stages of maturation. (B) Sections of oviducts and uteri.

**FIGURE 5:**
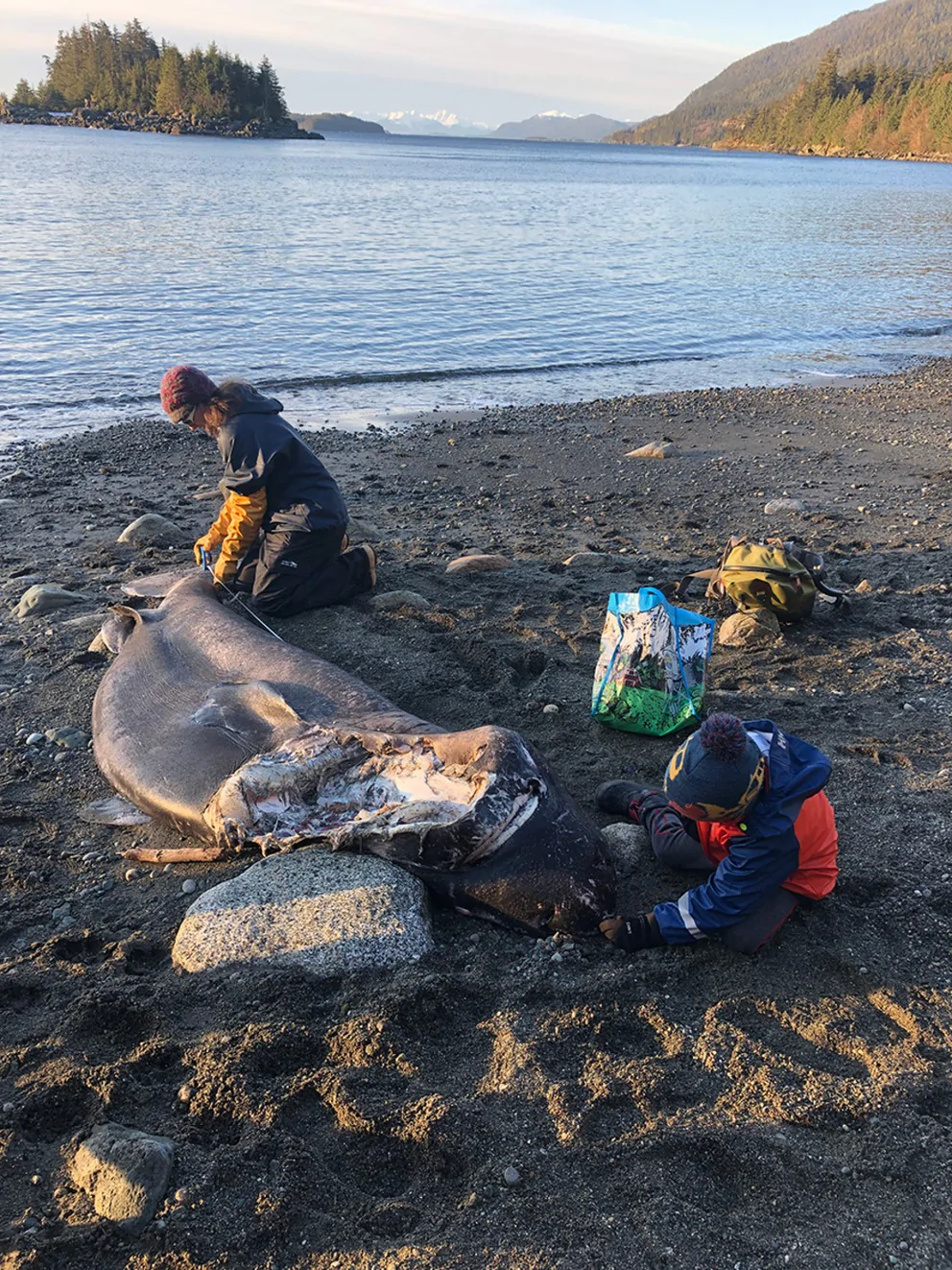
Body of stranded Pacific sleeper shark ( *Somniosus pacificus* ) found in Sunshine Cove, with significant trauma to the pharynx region.

**FIGURE 6:**
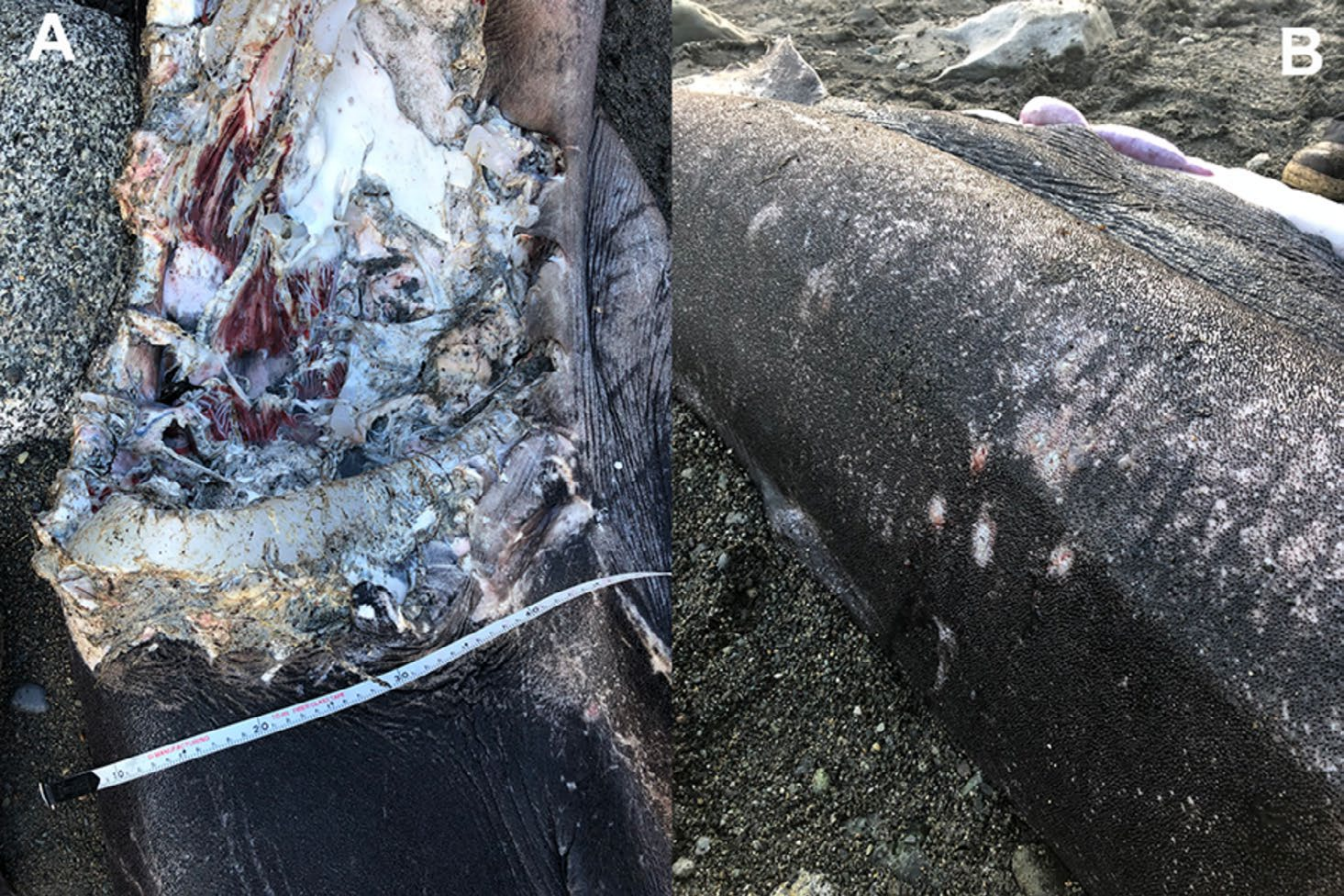
External trauma observed on the Pacific sleeper shark ( *Somniosus pacificus* ) found in Sunshine Cove. (A) Pharynx region. (B) tooth marks on the left abdomen.

**FIGURE 7:**
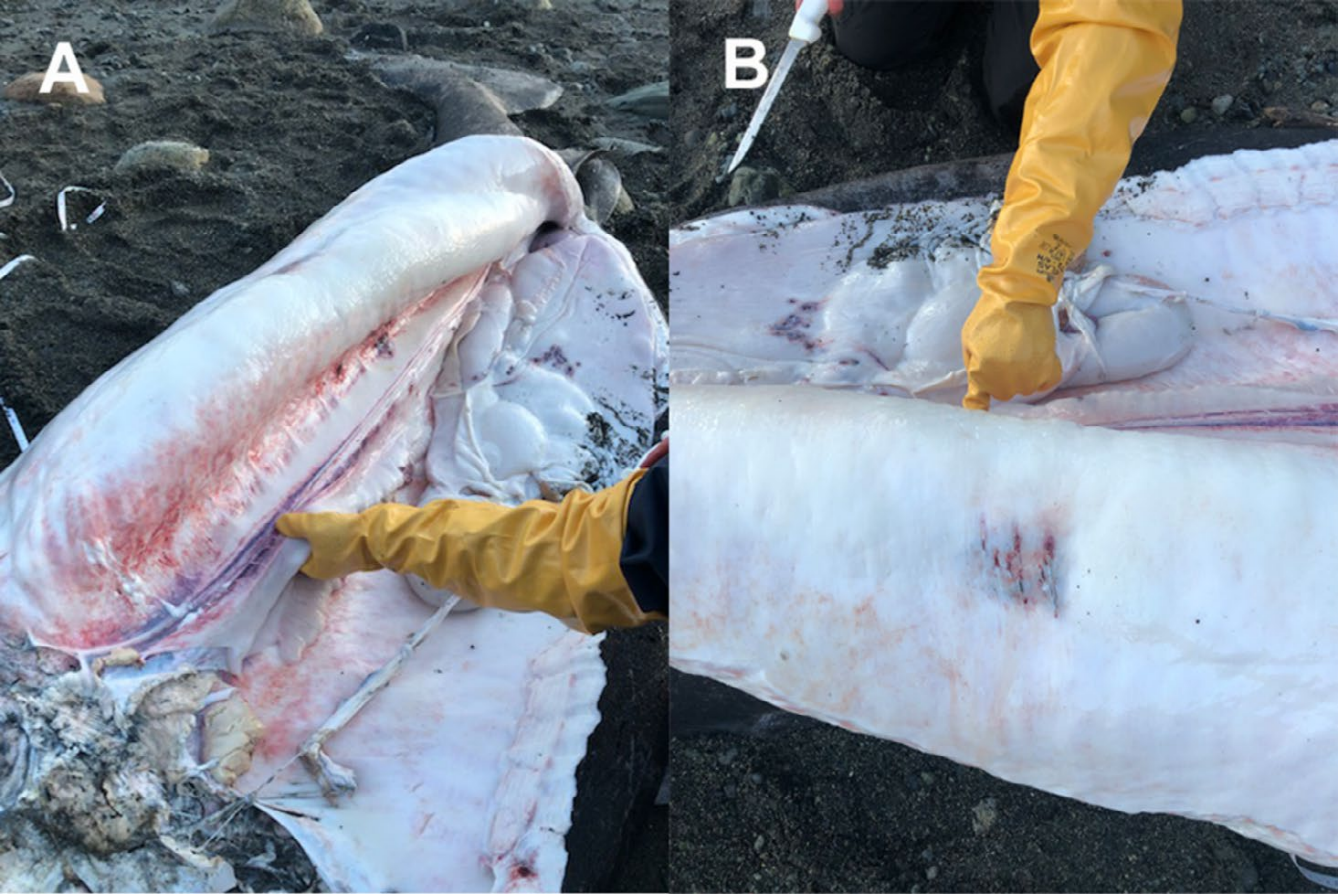
(A) Internal bruising on abdominal wall of the Pacific sleeper shark ( *Somniosus pacificus* ) found in Sunshine Cove. (B) Internal puncture wounds and bruising on the left ventral side.

Based on the observations, the injuries suggest that predation, likely by a killer whale, was the cause of mortality for this shark. The puncture wounds are consistent with killer whale bite wounds, as was the removal of the liver. Other studies have reported similar wounds on sharks caused by killer whales (Towner et al. 2024; Pancaldi et al. 2024). The liver is a lipid‐rich, efficient food source that does not contain the elevated levels of trimethylamine N‐oxide (TMAO) found in the shark muscle (Treberg and Driedzic 2002) and is therefore likely more palatable. While it is possible that a large Steller sea lion could kill a small Pacific sleeper shark, given the size of the shark in this report and the magnitude of the injuries, Steller sea lion predation seems unlikely.

Killer whale predation on elasmobranchs is not uncommon. The offshore killer whale ecotype in the eastern North Pacific Ocean has been documented to feed on elasmobranchs to the extent that it causes severe wear on the teeth (Ford et al. 2011). This ecotype has also been noted to prey specifically on Pacific sleeper sharks. Killer whale predation on Pacific sleeper sharks was first reported in Dixon Entrance, British Columbia, Canada (Ford et al. 2011), and instances have since been anecdotally reported from Prince William Sound (M. Horning, pers. comm) and in more comprehensive studies of predation events in the eastern North Pacific (Wright et al. 2025). In those cases, the attacks were inferred based on the activity of the killer whales and recovered floating tissues, which were later genetically identified as Pacific sleeper shark. In our case, the shark body was able to be examined, which provided potential insights into the nature of the attack. It is most likely that a group of offshore killer whales was moving through the inside waters of Southeast Alaska and was responsible for this predation event. Offshore killer whales have been documented in the area (COSEWIC 2023; Dalheim et al. 2008) with anecdotal reported sightings of over 40 individuals (Happywhale 2026). Further, high‐density multiple predation events by offshore killer whales on Pacific sleeper sharks have been observed in localized areas with apparent high Pacific sleeper shark occurrence (Ford et al. 2011) and the area in which this predation event occured was well within the documented dive depths for offshore killer whales (Schorr et al. 2022).

The mechanism of the predation event may be learned behavior, or cultural transmission (Amelot et al. 2022). In observed killer whale predation events on whale sharks ( *Rhincodon typus* ), the killer whales rammed the pelvic area of the sharks until the liver became accessible (Pancaldi et al. 2024). Conversely, in South Africa, pods of killer whales have been observed tearing open white sharks ( *Carcharodon carcharias* ) at the pectoral girdle to remove the liver (Towner et al. 2023). In one report, also in South Africa, a lone killer whale was responsible for the predation event of a white shark while another was observed nearby not participating (Towner et al. 2024). Yet, the non‐participating killer whale had been implicated in previous white shark predation events (Towner et al. 2023, 2024). Higuera‐Rivas et al. (2025) reported on a pod of killer whales observed in the Gulf of California that may be specialist elasmobranch predators, based on observed repeated attack methods and food sharing. To date, there have been no direct observations of similar predation events of killer whales on sharks in Alaska, but the observations reported here suggest biting and ramming behaviors with the liver being accessed through the area between the jaw and gills, accompanied by documented food sharing in some observations (Ford et al. 2011).

The relationship between marine mammals and Pacific sleeper sharks in Alaska is complex and difficult to observe. Given the vastness of Alaska waters and the low encounter probability, it is plausible that predation events by Steller sea lions and offshore killer whales on Pacific sleeper sharks could be a source of natural mortality that has not been evaluated. On at least two accounts, beach walkers in the Juneau area have reported finding severely decomposed Pacific sleeper sharks. In one case, the liver was associated with a Pacific sleeper shark carcass but detached from the gut cavity; in another case, only large, loose pieces of liver were found, which, based on size and coloration, appeared to be from a Pacific sleeper shark (C. Tribuzio, unpublished data). Such observations suggest that predation events such as these may be relatively common. Predation events could possibly change the localized behavior of sharks in the area, as observed in white sharks around the Farallon Islands (Reeves et al. 2026). Tagging data suggest that Pacific sleeper sharks residing in the inside waters of Southeast Alaska and within other embayments may remain in those areas for many years (C. Tribuzio unpublished data, Bishop et al. 2026), but fine‐scale and short‐term residency may still be altered by predation events such as these. Marine mammal activity may positively influence localized abundances of Pacific sleeper sharks as well. In the Aleutian Islands, transient killer whales predate upon migrating whales and stash the carcasses, resulting in greater concentrations of Pacific sleeper sharks around an easily scavenged food source (Barrett‐Lennard et al. 2011). Offshore killer whales have been observed in the same areas of the Aleutian Islands (Dalheim et al. 2008), but predation events in that region have not been reported.

While direct observations of predation events are rare and difficult to observe, reports and observations are crucial to understanding the ecological role of species within the Alaska marine ecosystem and informing the data‐limited population assessments. The Pacific sleeper shark serves as a large predator, a scavenger, and prey, filling many roles in the Alaska ecosystem. The deep fjords of southeastern Alaska may offer a depth refuge for Pacific sleeper sharks; however, evidence suggests that the sharks remain susceptible to predation by marine mammals.

Changes in the populations of mammalian predators may impact the frequency of predation events. The Eastern stock of Steller sea lions, which ranges from southeastern Alaska to California, has been increasing from 1990 to 2022 (Young et al. 2024). However, the Western stock of Steller sea lions, which ranges from the Gulf of Alaska to Japan, remains well below historic population levels (Young et al. 2024). The population trend of North Pacific offshore killer whales is unknown but believed to be stable (COSEWIC 2023). Documenting predation events on Pacific sleeper sharks is the first step to improving the understanding of the ecological roles the sharks have in the ecosystem and identifying potential impacts on their populations.

Monitoring Pacific sleeper sharks to better understand their ecological role is difficult, due to the vast expanse of Alaska waters and resource limitations. Leveraging monitoring of marine mammals can potentially act as a primary source of ecological information to inform the assessment of Pacific sleeper sharks (e.g., Dalheim et al. 2008, Ford et al. 2011, this report). Individual reports such as these are highly valuable to data‐limited assessments, especially when combined with citizen science, education, and outreach. While it is not possible to draw conclusions about the broader impact of this type of behavior at this time, these observations add to a growing body of knowledge. This information will be incorporated into population simulations for the Pacific sleeper shark and be used to inform harvest advice for fishery managers. Given the uncertainty in the natural mortality rates and likely slow replacement rates of Pacific sleeper sharks, a precautionary approach must be applied when assessing threats to this population.

## Author Contributions

**Cindy A. Tribuzio:** conceptualization (equal), data curation (equal), formal analysis (equal), investigation (equal), visualization (equal), writing – original draft (equal), writing – review and editing (equal). **John Moran:** conceptualization (equal), formal analysis (equal), investigation (equal), methodology (equal), writing – original draft (equal), writing – review and editing (equal).

## Funding

This research was funded through operational funds at the Alaska Fisheries Science Center, National Marine Fisheries Service, NOAA.

## Conflicts of Interest

The authors declare no conflicts of interest.

## Data Availability

All of the data supporting this study are reported in the manuscript. Any questions about the data or interpretations may be directed to the authors.
